# Mapping Drug-Resistant Tuberculosis Treatment Outcomes in Hunan Province, China

**DOI:** 10.3390/tropicalmed10010003

**Published:** 2024-12-24

**Authors:** Temesgen Yihunie Akalu, Archie C. A. Clements, Zuhui Xu, Liqiong Bai, Kefyalew Addis Alene

**Affiliations:** 1 School of Population Health, Faculty of Health Sciences, Curtin University, Perth, WA 6102, Australia; kefyalew.alene@curtin.edu.au; 2 Geospatial and Tuberculosis Research Team, Telethon Kids Institute, Perth, WA 6009, Australia; a.clements@qub.ac.uk; 3 Institute of Public Health, College of Medicine and Health Sciences, University of Gondar, Gondar 196, Ethiopia; 4 School of Biological Sciences, Queen’s University of Belfast, Belfast BT7 1NN, UK; 5 Xiangya School of Public Health, Central South University, Changsha 410078, China; xuzuhui@126.com; 6 TB Control Institute of Hunan Province, Changsha 410004, China; liqiong99@126.com

**Keywords:** mapping, drug-resistant tuberculosis, Hunan province

## Abstract

Background: Drug-resistant tuberculosis (DR-TB) remains a major public health challenge in China, with varying treatment outcomes across different regions. Understanding the spatial distribution of DR-TB treatment outcomes is crucial for targeted interventions to improve treatment success in high-burden areas such as Hunan Province. This study aimed to map the spatial distribution of DR-TB treatment outcomes at a local level and identify sociodemographic and environmental factors associated with poor treatment outcomes in Hunan Province, China. Methods: A spatial analysis was conducted using DR-TB data from the Tuberculosis Control Institute of Hunan Province, covering the years 2013 to 2018. The outcome variable, the proportion of poor treatment outcomes, was defined as a composite measure of treatment failure, death, and loss to follow-up. Sociodemographic, economic, healthcare, and environmental variables were obtained from various sources, including the WorldClim database, the Malaria Atlas Project, and the Hunan Bureau of Statistics. These covariates were linked to a map of Hunan Province and DR-TB notification data using R software version 4.4.0. The spatial clustering of poor treatment outcomes was analyzed using the local Moran’s I and Getis–Ord statistics. A Bayesian logistic regression model was fitted, with the posterior parameters estimated using integrated nested Laplace approximation (INLA). Results: In total, 1381 DR-TB patients were included in the analysis. An overall upward trend in poor DR-TB treatment outcomes was observed, peaking at 14.75% in 2018. Deaths and treatment failures fluctuated over the years, with a notable increase in deaths from 2016 to 2018, while the proportion of patients lost to follow-up significantly declined from 2014 to 2018. The overall proportion of poor treatment outcomes was 9.99% (95% credible interval (CI): 8.46% to 11.70%), with substantial spatial clustering, particularly in Anxiang (50%), Anren (50%), and Chaling (42.86%) counties. The proportion of city-level indicators was significantly associated with higher proportions of poor treatment outcomes (odds ratio (OR): 1.011; 95% CRI: 1.20 December 2024 001–1.035). Conclusions: This study found a concerning increase in poor DR-TB treatment outcomes in Hunan Province, particularly in certain high-risk areas. Targeted public health interventions, including enhanced surveillance, focused healthcare initiatives, and treatment programs, are essential to improve treatment success.

## 1. Background

Tuberculosis (TB) remains a leading cause of morbidity and mortality worldwide, particularly in low- and middle-income countries. The emergence of drug-resistant tuberculosis (DR-TB) has worsened the global TB crisis [1], presenting significant challenges to public health systems [2]. Despite advances in TB treatment, the treatment outcomes for DR-TB patients remain suboptimal, with high rates of treatment failure, death, and loss to follow-up (LTFU) [3].

China is among the countries with the highest burden of DR-TB, contributing significantly to the global incidence [4]. Within China, Hunan Province has experienced a growing public health challenge due to the increasing incidence of DR-TB [5]. The province, located in central China, is characterized by a mix of urban and rural areas with diverse sociodemographic and environmental conditions that may influence TB transmission and treatment outcomes [6]. However, the spatial distribution of DR-TB treatment outcomes and the associated risk factors in Hunan Province remain poorly understood [7]. The province has seen a significant burden of DR-TB in recent years, making it a critical area for public health research and intervention.

Spatial analysis of disease outcomes, particularly in a geographically and socioeconomically diverse region such as Hunan, is critical to understanding the underlying factors that contribute to poor treatment outcomes [8]. Identifying high-risk areas where treatment success is particularly low can inform targeted public health interventions and resource allocation, ultimately improving the management of DR-TB.

Ecological factors, including socioeconomic, healthcare, and environmental conditions, play a critical role in shaping the spatial distribution of poor treatment outcomes among DR-TB patients [9]. Socioeconomic disparities, such as poverty, low education levels, limited healthcare access, and delayed diagnosis, hinder treatment adherence [10,11], while rural areas face infrastructure gaps and sociocultural barriers, and urban overcrowding increases TB transmission risks [12,13]. Substance abuse further disrupts adherence and compounds vulnerabilities [14]. Environmental factors like air pollution, seasonal changes, and humidity impair recovery, while migration and mobility disrupt treatment continuity. Social stigma and limited community support reduce adherence, and geographic features like mountainous terrain and climatic variability pose logistical challenges to healthcare access [15].

Understanding the complex interplay of these ecological factors with the spatial distribution of DR-TB treatment outcomes is essential for developing effective strategies to combat DR-TB. This study aims to address these knowledge gaps by mapping the spatial distribution of DR-TB treatment outcomes in Hunan Province and identifying the sociodemographic, economic, healthcare, and environmental factors associated with poor treatment outcomes.

## 2. Methods and Materials

Study setting: This study was conducted in Hunan Province, located in central China. The province has a population of approximately 66.4 million people and is subdivided into 14 prefecture-level cities and 105 counties [16]. Hunan’s diverse landscape, ranging from mountainous areas to fertile plains, along with its varied climate, provides a unique setting to explore the spatial distribution of DR-TB treatment outcomes.

### 2.1. DR-TB Treatment Outcomes

In Hunan Province, DR-TB patients were treated with an individualized treatment regimen lasting 18 to 24 months, which included a combination of injectable drugs (Amikacin, Kanamycin, and Capreomycin), a quinolone (Levofloxacin, Gatifloxacin, and Moxifloxacin), and other medications such as Para-Aminosalicylic Acid, Prothionamide, Pyrazinamide, Clarithromycin, Cycloserine, or Ethambutol. In addition to medication, patients received psychological and social support and underwent regular sputum smear and culture tests, with medication refills provided as needed. Recently, Hunan Province has implemented the WHO-recommended shorter treatment regimens for drug-resistant tuberculosis (lasting 9 to 12 months) [17]. Upon completion of treatment, the outcomes were classified as either poor (death, treatment failure, or LTFU) or good (cure or treatment completion).

A patient was classified as cured if they completed treatment without evidence of failure and had at least three consecutive negative cultures taken at least 30 days apart. Those who completed treatment without evidence of failure but lacked bacteriological confirmation were classified as having completed treatment. Death was defined as occurring during DR-TB treatment, regardless of the cause. Treatment failure was identified if a patient permanently discontinued treatment, changed at least two DR-TB drugs due to adverse reactions, experienced bacteriological reversion, or failed to achieve culture conversion by the end of the intensive phase. LTFU was defined as not starting or interrupting treatment for at least two consecutive months [17].

### 2.2. Study Population

This study included all the DR-TB patients who were enrolled and completing treatment between 2013 and 2018. Patients residing outside the hospital’s catchment area, including those from adjacent provinces, were excluded from the study.

### 2.3. Data Source and Collections

This study analyzed all the patients diagnosed with DR-TB and treated in Hunan Province between 2013 and 2018, excluding those reporting adverse effects only, those transferred out, and individuals whose diagnoses changed. Data were sourced from the TB Control Institute of Hunan Province. The treatment outcomes were categorized as cure, completion, death, loss to follow-up, and treatment failure, based on the definitions provided in the supplementary files (Supplementary File S1). These data involved patients with DR-TB as part of routine TB surveillance. Data for the treatment outcomes were aggregated at the county level to identify the number of patients with poor treatment outcomes per county.

Demographic and socioeconomic data, including the male population percentage, average gross domestic product (GDP), birth rate and mortality rate, and city-level indicators, were obtained from the Hunan Bureau of Statistics. Healthcare access metrics, such as the number of health institutions, medical personnel, contraceptive utilization rate, and hospital beds per 10,000 population, were also included. Climatic variables (mean temperature, precipitation, radiation, and wind) were derived from the global climatic database [18], while data on the travel time to cities were sourced from the Malaria Atlas Project [19].

The spatial data integration and analysis were performed using R software (version 4.4.0) and ArcGIS Pro (version 3.3). Administrative boundary shapefiles for Hunan Province were used to process the spatial data. Spatially continuous covariates were summarized at the county level using the ArcGIS Pro tool, and unwanted areas were removed with the “crop” function from the raster package to enhance the visualization. Multiple raster areas were stacked into a single object for simplified analysis and multivariable regression. Resampling was conducted to ensure consistency in the resolution and extent, improving the quality of the spatial data analysis.

### 2.4. Descriptive Non-Spatial Analysis

The prevalence of poor treatment outcomes was calculated by summing the number of deaths, treatment failures, and LTFUs, and then dividing by the total number of enrolled DR-TB cases in each county. Initially, a univariate analysis was performed, with the proportion of poor DR-TB treatment outcomes as a response variable and various geographical covariates as the independent variables. Covariates with a *p*-value of <0.2 were selected for inclusion in the final model, and multicollinearity was assessed using a variance inflection factor (VIF) threshold of >5, leading to the exclusion of variables with VIF values above this threshold. City-level indicators (%) refer to the proportion of a city or urbanized area based on the infrastructure, population density, and urban characteristics like residential, commercial, and industrial zones.

### 2.5. Descriptive Spatial Analysis

Global Moran’s I statistics were used to investigate the spatial clustering and spatial autocorrelation of poor treatment outcomes among DR-TB patients. The local spatial dependency was assessed using the local Moran’s I, with values ranging from –1 to +1 indicating distinct spatial patterns. The clustering was further evaluated using Anselin local Moran’s I and Getis–Ord statistics. Positive Z-scores indicated clustering of high values (hotspots), while negative Z-scores indicated clustering of low values (cold spots). The county-level clustering and hotspot detection were conducted using ArcGIS Pro software.

### 2.6. Bayesian Spatial Analysis

Three different models were constructed, and parameter estimation was performed using integrated nested Laplace approximation (INLA). The models included a spatially unstructured model (Model I), a spatially structured model with covariates (Model II), and a combined spatially structured and unstructured model with covariates (Model III).

The general formula used in the model construction was specified as follows:

*Y_i_*~*Binomial*(*n_i_*, *p_i_*)

where:

- *Y_i_* represents the number of poor treatment outcomes among drug-resistant tuberculosis (DR-TB) patients in county *i*,
- *n_i_* is the total number of DR-TB patients in county *i*,
- *p_i_* is the probability of poor treatment outcomes among DR-TB patients in county
- To model the probability p*_i_*, the logit link function was applied: Logit (P*_i_*) = **α** + $\sum_{N}$ ***βn*** ∗ ***Xn***, ***i*** + ***Ui*** + ***Vi***, where represents the probability of poor treatment outcomes among DR-TB patients in county *i*; α is the intercept; ∑*_N_ βn* ∗ *Xn*, *i* denotes the matrix of independent county-specific covariates (X, i.e., proportions of males, contraceptive rate, number of health institutions, and city-level factors (%)) measured at each county *i*, multiplied by their coefficients ***β***; U*_i_* represents unstructured random effects (error term); and V*_i_* represents spatially structured random effects. The model selection was guided by the deviance information criterion (DIC), with the model with the lowest DIC being identified as having the best fit.

## 3. Results

### 3.1. Sociodemographic Characteristics of DR-TB Patients

Between 2013 and 2018, 2274 DR-TB cases were reported to the Hunan TB control program from 103 counties. Of these, the treatment outcome was assessed for 1384 patients, while the remaining 890 were excluded because they were either still on treatment or had been transferred out. Additionally, three DR-TB cases from outside Hunan Province were excluded from the analysis. This left a final sample of 1381 DR-TB patients for analysis. Among these patients, more than three-fourths (77.62%) were male, and 1083 (78.42%) were farmers by occupation. The median age of the DR-TB patients was 51 years, with an interquartile range (IQR) of 70 years. Most of the patients, 1091 (79.00%), were new cases. Nearly one-third of the DR-TB cases, 400 (28.96%), were enrolled in 2018. Of the 1381 DR-TB patients, 138 (9.99%) experienced poor treatment outcomes (Table 1).

### 3.2. Trends of Poor DR-TB Treatment Outcomes

**Figure 1:**
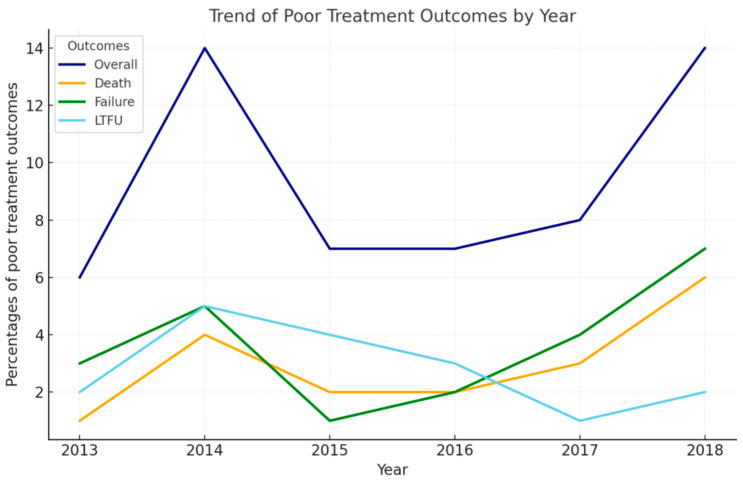
Trend analysis of poor treatment outcomes, deaths, treatment failures, and LTFU among DR-TB patients in Hunan Province, 2013–2018.

The trend analysis from 2015 to 2018 in Hunan Province showed fluctuations in poor treatment outcomes among DR-TB patients. The overall percentage of poor treatment outcomes peaked at 13.86% in 2014, followed by a decline and a sharp increase to 14.75% in 2018. Deaths and treatment failures showed similar patterns, with deaths notably increasing to 4.75% by 2018 and treatment failures reaching 9.5%. In contrast, the proportion of patients LTFU dropped steadily from 1.98% in 2014 to just 0.5% in 2018, indicating significant improvements in patient retention over the study period (Figure 1).

### 3.3. Proportions of Poor DR-TB Treatment Outcomes

**Figure 2:**
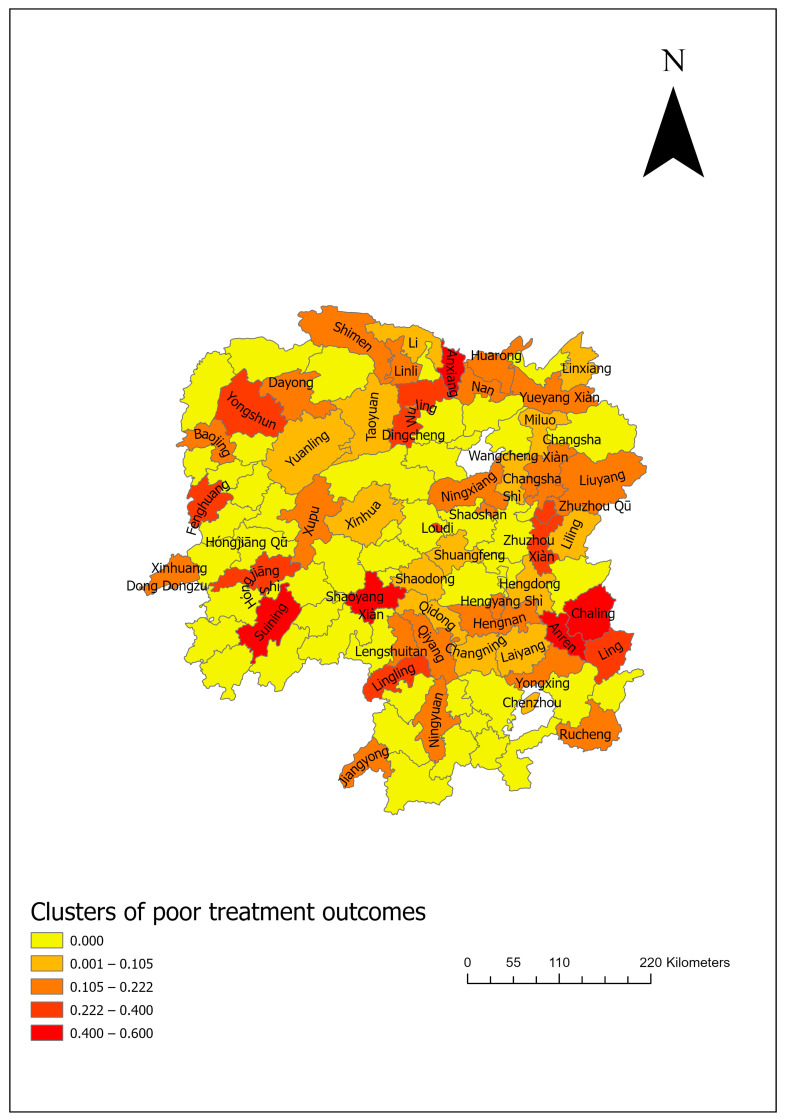
Spatial distribution of poor treatment outcomes by county in Hunan Province, 2013–2018.

The overall proportion of poor DR-TB treatment outcomes in Hunan Province was 9.99%, with a 95% CI of 8.46% to 11.70%. Notably, higher proportions of poor treatment outcomes were observed in Anxiang (50%), Anren (50%), and Chaling (42.86%) counties (Figure 2).

### 3.4. Spatial Clustering of Poor DR-TB Treatment Outcomes

**Figure 3:**
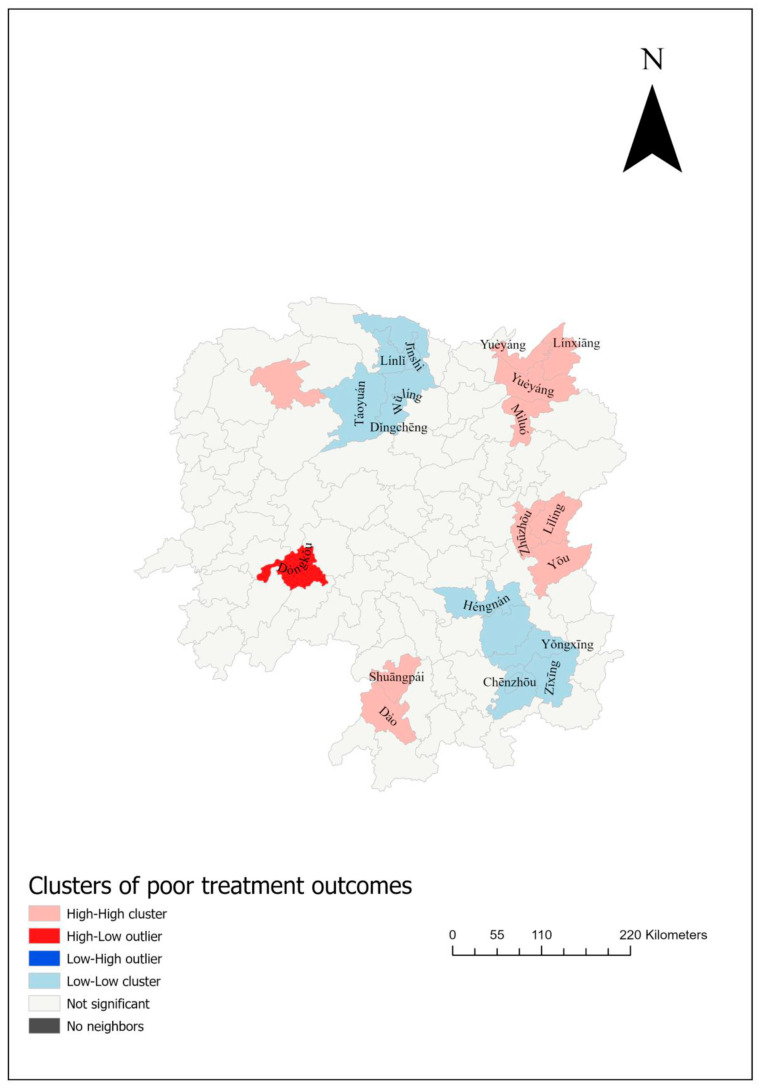
Spatial clustering of poor treatment outcomes among DR-TB patients in Hunan Province, 2013–2018, based on the Anselin local Moran’s I.

The spatial distribution of poor DR-TB treatment outcomes was notably clustered at the county level (Supplementary Figure S1), as confirmed by a Moran’s I of 0.52 (*p* < 0.0001; Z 6.986). Significant high–high clusters of poor treatment outcomes were identified in Xintian, Huáróng, Yuánjiāng, Ningyuan, Pingjiang, Xiangyin, Yueyang Xiàn, Sangzhi, Liling, and Youxian, all of which bordered Hubei and Jiangxi (Figure 3).

This map illustrates the spatial clusters of poor DR-TB treatment outcomes in Hunan Province. The red areas indicate high–high clusters, where similar high-proportion counties surround counties with high proportions of poor treatment outcomes. The red areas show high–low outliers, where high proportions of poor treatment outcomes are surrounded by counties with lower proportions.

**Figure 4:**
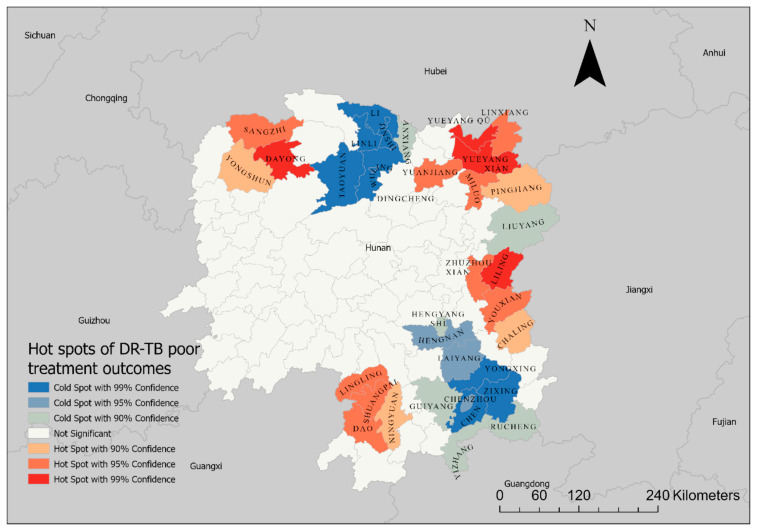
Spatial clustering of poor DR-TB treatment outcomes in Hunan Province, 2013–2018, based on the Getis–Ord G-statistics.

Based on the Getis–Ord G statistics, hotspots and high clustering were detected in the border regions of Hunan Province, particularly in the northeast, northwest, east, and southwest parts of the province (Figure 4). This map shows the hotspots and cold spots of poor DR-TB treatment outcomes in Hunan Province, with varying levels of statistical confidence. The red areas represent hotspots, where there is a high concentration of poor treatment outcomes, with the intensity of the red color indicating the confidence level (dark red for 99% confidence and lighter red for 90–95% confidence). These hotspots are primarily located in the eastern and western parts of the province. The blue areas indicate cold spots, where poor treatment outcomes are less concentrated, again with varying confidence levels (dark blue for 99% confidence and lighter blue for 90–95% confidence). These cold spots are clustered in the central part of the province. Areas not shaded represent regions with no significant clustering of poor treatment outcomes.

### 3.5. Ecological-Level Factors Associated with Spatial Clustering of Poor Treatment Outcomes

The best-fitting model, based on the lowest DIC value from the multivariate Bayesian regression models, was Model II, which contained the spatially structured random effect and covariates. This model indicated that the proportions of poor DR-TB treatment outcomes were significantly associated with the proportion of city-level indicators (%) (OR: 1.011; 95% CRI: 1.001–1.035) (Table 2 and Table 3).

**Figure 5:**
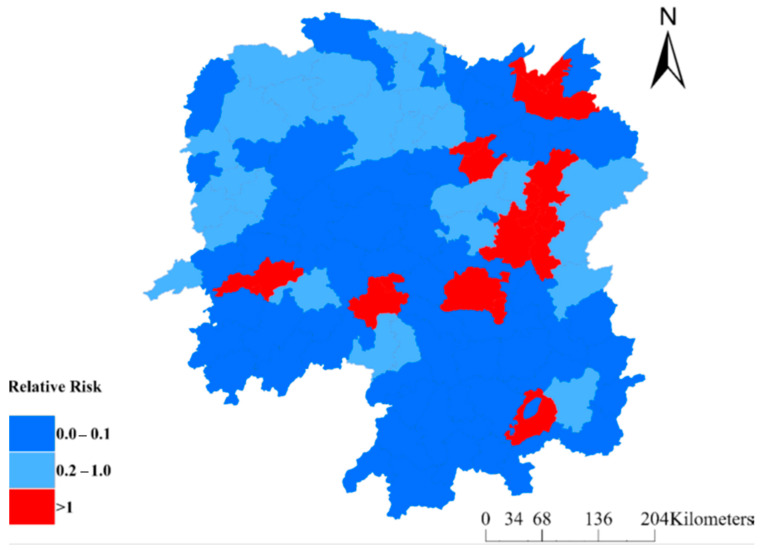
Posterior mean of the spatially structured random effects for poor treatment outcomes among drug-resistant tuberculosis patients in Hunan Province, 2013–2018.

After accounting for ecological-level factors, the posterior mean of the spatially structured random effects revealed clustering within the province (Figure 5), suggesting significant county-level heterogeneity in DR-TB that remains unexplained by the included factors.

Figure 5 shows the relative risk of poor DR-TB treatment outcomes across Hunan Province, with red areas indicating counties at higher risk (relative risk > 1) and blue areas representing lower-risk regions. The red counties are hotspots with a significantly higher occurrence of poor treatment outcomes. Conversely, the blue regions, particularly those that are dark blue, have a lower-than-expected occurrence of poor outcomes.

## 4. Discussion

This study is the first to investigate the spatial distribution and ecological factors associated with poor DR-TB treatment outcomes in Hunan Province. The findings show significant spatial clustering of poor treatment outcomes, with certain counties, such as Anxiang, Anren, and Chaling, reporting notably higher proportions, where the rates exceeded 40%. These results indicate regional disparities in DR-TB management and the need for targeted public health strategies in these areas. Similar studies conducted in other countries on the spatial clustering of poor treatment outcomes among DS-TB cases have reported comparable findings, with spatial clustering observed in China, India, and Ethiopia [20]. Several factors may explain the observed clustering of poor treatment outcomes among DR-TB patients in Hunan Province. One possible explanation is the prevalence of resistant TB strains, such as Beijing strains, which are associated with higher transmission rates, increased virulence, and a greater likelihood of poor treatment outcomes in certain counties. This localized spread may contribute to the clustering observed in neighboring communities. Environmental factors, including overcrowding and poverty, increase the risk of poor treatment outcomes [21]. Socioeconomic disparities, along with cultural beliefs and practices, may also play a significant role by influencing awareness of DR-TB, treatment-seeking behavior, and adherence to prescribed treatments [22]. The particularly high burden of poor treatment outcomes in the eastern and southwestern parts of the province could be attributed to limited access to healthcare and the poor quality of healthcare services in these regions.

The lack of clustering of poor treatment outcomes among DR-TB patients in most counties of Hunan Province could be attributed to improved healthcare access, effective public health interventions, equitable resource distribution, and fewer socioeconomic disparities in these regions. Additionally, the geographic and population distribution and lower disease severity may contribute to better treatment outcomes. In some cases, random variability or smaller sample sizes might also explain the absence of clustering. Further analysis is needed to better understand these factors and their influence on the spatial distribution of treatment.

Our study showed an increasing trend in poor treatment outcomes among DR-TB patients in the Hunan Province in recent years. Several factors may contribute to this rise. The number of DR-TB cases diagnosed and initiated treatment has been growing, partly due to advancements in diagnostic services, such as the introduction of GeneXpert technology [23]. This increase might be attributed to the higher sensitivity of GeneXpert in detecting MTB/RIF, as reported in previous studies [24,25,26]. However, this also presents challenges, as higher detection rates may lead to increased mortality and treatment failure if the health system is not equipped to manage the growing caseload. Issues such as inadequate healthcare infrastructure, staffing shortages, and suboptimal infection control practices can exacerbate these outcomes. On the other hand, a previous systematic review found that GeneXpert did not affect treatment outcomes in TB patients [27], while improved diagnostics are crucial, they must be complemented by robust health system support to improve patient outcomes.

Conversely, there has been a decrease in LTFU over time in Hunan Province, attributed to the enhanced implementation of DOTs and new patient-tracking mechanisms. For example, Hunan Chest Hospital has introduced a specialized system to notify and support patients who have not enrolled or who have missed appointments [28]. Additionally, Hunan Province has adopted the WHO-recommended shorter DR-TB treatment regimens (9–12 months), which are particularly effective in reducing LTFU among patients facing challenges like out-migration and economic hardship [29].

We found that the city-level percentage was significantly associated with the spatial clustering of poor treatment outcomes among DR-TB patients. Despite the higher level of healthcare access and infrastructure in urban areas, our study found a negative association between urbanization and poor treatment outcomes among DR-TB patients in Hunan Province. Several factors contribute to this finding. Cities attract migrants from rural areas seeking better job opportunities, which can disrupt their treatment continuity and strain healthcare resources due to staff shortages and high patient turnover [30]. This influx also introduces competing health priorities, leading to delays in diagnosis and treatment initiation, which negatively impacts treatment outcomes. Additionally, migrant populations often face barriers to healthcare access such as high costs, cultural differences, and language barriers, which hinder timely diagnosis, treatment initiation, and adherence, which in turn affect treatment outcomes [31]. Certain urban industries and occupations may expose TB patients to environmental hazards that can complicate treatment outcomes [32].

Poor food quality and insufficient quantity significantly affect treatment outcomes among DR-TB patients by compromising their nutritional status, which is critical for immune function and recovery [33,34]. Malnutrition weakens the body’s ability to fight TB, increases susceptibility to side effects of medications, and prolongs the time to recovery [35,36]. Inadequate nutrition may also reduce the effectiveness of TB drugs by impairing drug metabolism and absorption [37]. Nutritional deficiencies, such as low levels of vitamins A, D, and zinc, further impair immunity and healing [38], leading to higher rates of treatment failure, prolonged infectiousness, and increased mortality [39]. However, a lack of data on food quality and quantity in the population under study limits us from conducting further analysis.

Our study has some important limitations. Firstly, the ecological nature of the study design limits our ability to establish causation between poor treatment outcomes and the covariates examined. Secondly, we did not include important clinical variables that could significantly affect poor treatment outcomes, such as HIV/AIDS status, comorbidities (e.g., diabetes mellitus), and behavioral factors (e.g., smoking and alcohol consumption), due to the lack of available data on these variables. This study also lacks data regarding the treatment outcomes during and after COVID-19. Further studies are required to explore the effect of the COVID-19 pandemic on the spatial distribution of poor DR-TB treatment outcomes. Moreover, the predominance of a single ethnicity in the dataset could introduce bias into the final model. However, we believe the potential impact on the results is minimal in this context, as our analysis focuses primarily on variables less influenced by ethnicity, such as environmental, clinical, or geographical factors.

## 5. Conclusions

Spatial clustering was detected in the eastern and some southwestern parts of Hunan Province. The annual incidence rate of poor treatment outcomes increased over time. Our findings underscore the need for strengthened public health strategies, including enhanced surveillance, specialized healthcare initiatives, and tailored treatment programs. Interventions should focus on regions with high incidence rates and identified clusters of poor outcomes to improve the overall success of DR-TB treatment. By targeting high-risk areas and addressing specific types of resistance, it may be possible to reduce disparities and improve the effectiveness of DR-TB treatment strategies in Hunan Province.

## Figures and Tables

**Table 1:** Sociodemographic and clinical characteristics of DR-TB patients in Hunan Province, 2013–2018.

Variable	Number (%)
Gender	
Female	309 (22.38)
Male	1072 (77.62)
Median age (in years)	51 with IQR 77 years
Occupation	
Child or student	38 (2.75)
Gov’t employed	26 (1.88)
Farmers	1083 (78.42)
Housekeeping	104 (7.53)
Private employed	21 (1.52)
Retired	63 (4.56)
Other	46 (3.33)
Ethnicity	
Han	1310 (94.86)
Dong	7 (0.51)
Miao	20 (1.45)
Tujia	30 (2.17)
Yao	9 (0.65)
Other *	5 (0.35)
Severely ill	
No	1302 (94.28)
Yes	79 (5.72)
Treatment category	
New	1091 (79.00)
Re-treatment	290 (21.00)
Patient source	
Health check	2 (0.14)
Recommend consultation due to symptoms	13 (0.43)
Referral	489 (35.41)
Seeking consultation due to symptoms	416 (30.12)
Tracing	455 (32.95)
Other	6 (0.43)
Year of DR-TB enrollment	
2013	83 (6.01)
2014	101 (7.31)
2015	178 (12. 98)
2016	314 (22.74)
2017	305 (22.09)
2018	400 (28.96)
Treatment outcome	
Good treatment outcome	1243 (90.01)
Poor treatment outcome	138 (9.99)

Other * Bai, Mongolian, Tibetan, and Zhuang.

**Table 2:** Univariate model for poor treatment outcomes at the county level in Hunan Province, China, 2013–2018.

Independent Variables	Coefficient (95% CI)	*p*-Value
Male proportion	1.014	0.033
City level (%)	1.017	<0.001
GDP (CNY 10,000)	1.001	0.009
Number of hospital beds per 10,000 population	1.007	0.004
Number of health institutions per 10,000 population	0.908	0.002
Number of medical personnel per 10,000 populations	1.007	0.025
Average temperature (°C)	1.107	0.337
Average precipitation (mm)	1.011	0.9
Average radiation (kJ/m^2^/day)	1.016	0.862
Average wind (m/s)	0.119	0.898
Travel time/distance from health facilities	0.116	0.235

**Table 3:** Bayesian logistic regression model for poor DR-TB treatment outcomes by county in Hunan Province, China, 2013–2018.

Variables	Unstructured Model OR, Posterior Mean (95% CI)	Structured Model OR, Posterior Mean (95% CI)	Structured and Unstructured Model OR, Posterior Mean (95% CI)
Male proportion	0.992 (0.975–1.008)	0.992 (0.974–1.011)	0.992 (0.974–1.008)
City level (%)	1.018 (0.997–1.026)	1.011 (1.001–1.035)	1.011 (1.002–1.026)
Contraceptive rate	0.968 (0.932–1.006)	0.969 (0.932–1.008)	0.969 (0.932–1.006)
Number of the health institution	0.947 (0.882–1.011)	0.946 (0.878–1.019)	0.942 (0.877–1.012)
Intercept α	0.745 (−2.996–4.486)	0.641 (−3.241–4.492)	0728 (−3.035–4.492)
DIC	307.4492	304.4255	307.0945

## Data Availability

Data will be available upon request from the corresponding author.

## Supplementary Materials

The following supporting information can be downloaded at https://www.mdpi.com/article/10.3390/tropicalmed10010003/s1, Table S1: Incidence rate of DR-TB in Hunan Province at the county-level; Table S2: Type of drug, chemical structures, and classes of anti-drug-resistant tuberculosis treatment; Figure S1: Trend analysis of overall poor treatment outcomes in Hunan Province and high incidence counties (Anxiang, Anren, and Chaling County) among DR-TB patients in Hunan Province, 2013–2018.

## Abbreviations

CI: credible interval, DIC: deviance information criteria, DR-TB: drug-resistant tuberculosis, GDP: gross domestic product, GIS: geographical information criteria, INLA: integrated nested Laplace approximation, IQR: interquartile range, LTFU: loss to follow-up, OR: odds ratio, TB: tuberculosis, and VIF: variance inflation factor.
