# Supplementary material for: Mapping Drug-Resistant Tuberculosis Treatment Outcomes in Hunan Province, China
Source: Trop Med Infect Dis. 2024 Dec 24;10(1):3. doi: 10.3390/tropicalmed10010003 (PMC11769319; doi:10.3390/tropicalmed10010003)
Supplement: Supplementary file 1 [file tropicalmed-10-00003-s001.zip › tropicalmed-3262009-supplementary.pdf]

Supplementary files

Table S1: Incidence rate of DR-TB in Hunan Province at the county-level

County name	Incidence of DR-TB
Ānxiāng	0.5
Dǐngchēng	0.4
Hànshòu	0
Jīnshì	0
Línlǐ	0.14
Li	0.04
Shimen	0.167
Taoyuan	0.04
Wuling	0.2
Changsha Shì	0.18
Changsha Xiàn	0.11
Liuyang	0.2
Ningxiang	0.21
Wangcheng	0.03
Anren	0.5
Chenzhou	0.03
Guidong	0
Guiyang	0
Jiahe	0
Linwu	0
Rucheng	0.14
Yizhang	0
Yongxing	0.11
Zixing	0
Changning	0.06
Hengdong	0.06
Hengnan	0.17
Hengshan	0
Hengyang Shì	0.14
Hengyang Xiàn	0
Laiyang	0.08
Qidong	0.1
Chenxi	0
Hóngjiāng Qū	0.33
Hóngjiāng Shì	0.33
Huaihua	0
Huitong	0
Jing	0
Mayang Miao	0
Tongdao Dong	0
Xinhuang Dong Dongzu	0.14
Xupu	0.13
Yuanling	0.07
Zhijiang Dong Dongzu	0

Lengshuijang	0
Lianyuan	0
Loudi	0.27
Shuangfeng	0.11
Xinhua	0.06
Chengbu Miao	0
Daxiang	0
Dongkou	0
Longhui	0
Shaodong	0.04
Shaoyang Shì	0
Shaoyang Xiàn	0.6
Suining	0.5
Wugang	0
Xinning	0
Xinshao	0
Shaoshan	0.15
Xiangtan Shì	0
Xiangtan Xiàn	0
Xiangxiang	0
Baojing	0.2
Fenghuang	0.27
Guzhang	0
Huayuan	0
Jishou	0
Longshan	0
Luxi	0
Yongshun	0.3
Anhua	0
Nan	0.15
Taojiang	0
Yiyang Shì	0
Yuanjiang	0
Dao	0
Jianghua Yao	0
Jiangyong	0.14
Lanshan	0
Lengshuitan	0.22
Lingling	0.33
Ningyuan	0.11
Qiyang	0.19
Shuangpai	0
Xintian	0
Huarong	0.18
Linxiang	0.04
Miluo	0.04
Pingjiang	0
Xiangyin	0

Yueyang Qū	0
Yueyang Xiàn	0.18
Cili	0
Dayong	0.2
Sangzhi	0
Chaling	0.43
Liling	0.08
Ling	0.33
Youxian	0
Zhuzhou Qū	0.26
Zhuzhou Xiàn	0.33

Supplementary Figures

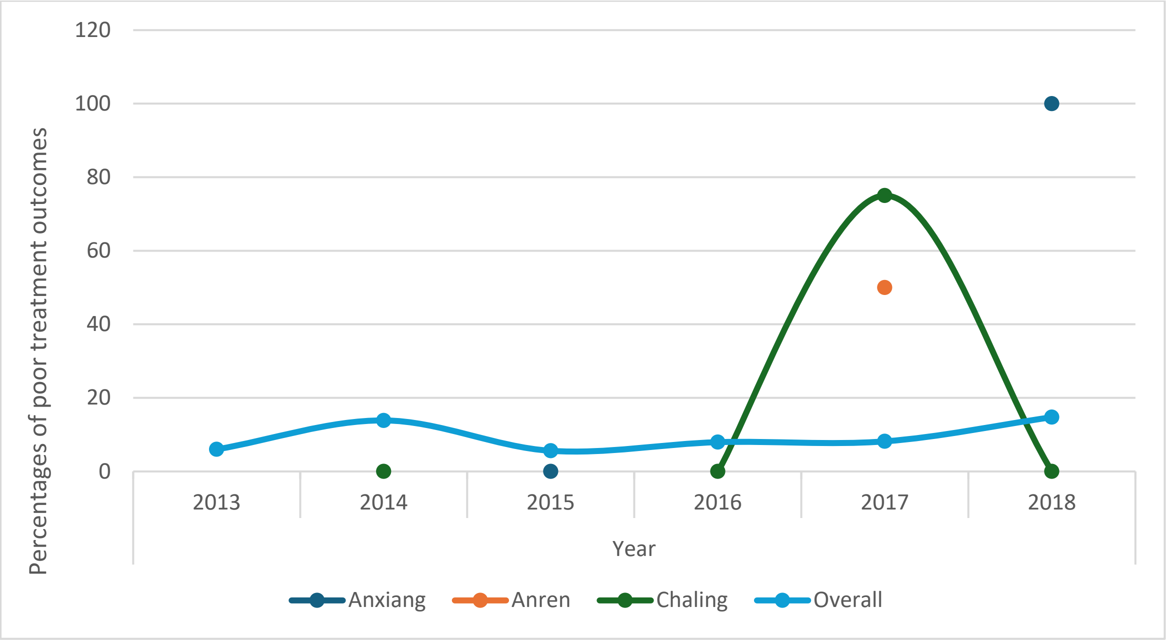

Figure S1: Trend analysis of overall poor treatment outcomes in Hunan Province and high incidence counties (Anxiang, Anren, and Chaling County) among DR-TB patients in Hunan Province, 2013-2018

Supplementary table S2:

Drug class	Drug name	Chemical structure	Level of resistance
Injectable Antibiotics	Amikacin	$C_{22}H_{43}N_5O_{13}$	High resistance in DR-TB strains
	Kanamycin	$C_{18}H_{36}N_4O_{11}$	Similar resistance level as Amikacin
	Capreomycin	C25H51N7O12	Moderate to high resistance
Quinolones	Levofloxacin	C18H20FN3O4	Resistance can develop rapidly
	Gatifloxacin	C18H20FN3O4	Some DR-TB Strains resistant
	Moxifloxacin	C21H24FN3O4	Resistance in specific DR-TB cases
Other Antibiotics	Para-aminosalicylic acid	C7H7NO3	Resistant in observed in chronic cases
	Prothionamide	C9H12N2S2	High resistance in some cases
	Pyrazinamide	C5H5N3O	Resistance varies; low to moderate
	Clarithromycin	C38H69NO13	Variable resistance; limited use in TB
	Cycloserine	C6H12N2O2	Moderate resistance, used sparingly
	Ethambutol	C10H24N2O2	Resistance can develop quickly
